# The difference of variation types between late-onset multiple acyl-CoA dehydrogenase deficiency patients carrying biallelic and single heterozygous variations in *ETFDH*: a systematic review and meta-analysis

**DOI:** 10.1186/s13023-025-03845-7

**Published:** 2025-06-18

**Authors:** Huiqiu Zhang, Jing Ma, Menghan Su, Junsen Zhao, Weisong Duan, Juan Wang, Dan Liu, Junhong Guo, Xueli Chang, Wei Zhang, Rongjuan Zhao

**Affiliations:** 1 Department of Neurology, First Hospital of Shanxi Medical University, No. 85, Jiefang South Street, Taiyuan, 030012 China; 2 Research Center for Neurological Diseases, Shanxi Medical University, Taiyuan, China; 3 First Clinical Medical College, Shanxi Medical University, Taiyuan, China; 4 Department of Neurology, Second Hospital of Hebei Medical University, Shijiazhuang, China

**Keywords:** Late-onset multiple acyl-CoA dehydrogenase deficiency, *ETFDH*, Single heterozygous variations, Variation types, Meta-analysis

## Abstract

**Background:**

Late-onset multiple acyl-CoA dehydrogenase deficiency (MADD) is an autosomal recessive disease chiefly caused by mutations in *ETFDH* gene. Mutations in the *ETFDH* gene lead to abnormal structure, impaired function, and increased degradation of ETFDH protein. However, it is not known why approximately 10% of patients carry single heterozygous variants in *ETFDH*. We speculate that different variation types (e.g., null variants and missense variants) partially account for the phenomenon.

**Methods:**

In this study, six databases were searched up until December 01, 2024. Studies describing late-onset MADD patients carrying *ETFDH* variations were included. The analyses focused on the differences in variation types, computational pathogenicity scores of missense variants, and clinical characteristics between patients with biallelic and single heterozygous variations (biallelic group vs heterozygous group).

**Results:**

Of the initially screened 3638 studies, 30 met the inclusion criteria, including 498 late-onset MADD patients with biallelic variations and 62 with single heterozygous variations in *ETFDH*. The relative frequency of patients carrying null variants was lower in the biallelic group (21%, 95% CI [16%-27%]) than that in the heterozygous group (34%, 95% CI [23%-48%]) (*P* = 0.044). Missense variants in the heterozygous group had stronger pathogenicity than those in the biallelic group, as reflected by the computational prediction tools, SIFT, PolyPhen-2 and metaRNN (*P* < 0.05). Patients carrying biallelic variations had a younger onset age and a higher level of serum creatine kinase at diagnosis (*P* < 0.05).

**Conclusions:**

Late-onset MADD patients carrying single heterozygous variations in *ETFDH* gene have distinct variation profiles and clinical severity compared to those harboring biallelic variations, which highlights the complexity of this disease.

**Supplementary Information:**

The online version contains supplementary material available at 10.1186/s13023-025-03845-7.

## Introduction

Multiple acyl-CoA dehydrogenase deficiency (MADD), also known as glutaric acidemia type II (GAII), is an autosomal recessive disorder affecting fatty acid, amino acid, and choline metabolism [1, 2]. The phenotypes of MADD can be classified into 3 categories: neonatal-onset form with congenital anomalies, neonatal-onset form without congenital anomalies, and late-onset form [3, 4]. Late-onset MADD, predominantly caused by *ETFDH* gene mutations, is the most common lipid storage myopathy [5]. *ETFDH* gene encodes electron transfer flavoprotein dehydrogenase (ETFDH), which transfers electrons from multiple acyl-CoA dehydrogenases to the respiratory chain [6]. As a result, functional loss of ETFDH affects fatty acid oxidation [7]. Concurrently, under the combined effects of fatty acid oxidation and riboflavin deficiency, the flavin adenine dinucleotide (FAD) pool is depleted. This depletion compromises the structural stability of the ETFDH protein, rendering it more susceptible to degradation, ultimately resulting in MADD [8]. In addition, electron transfer flavoprotein (ETF) genes (*ETFA* and *ETFB*), some riboflavin transporter genes (*SLC52A1*, *SLC52A2*, *SLC52A3*, and *SLC25A32*), FAD synthase gene (*FLAD1*), and coenzyme A synthase gene (*COASY*) were also reported as the causative genes for late-onset MADD [9–11].

Late-onset MADD is inherited in an autosomal recessive manner, and patients often carry compound heterozygous or homozygous mutations in *ETFDH* gene [5]. However, it is not known why approximately 10% of late-onset MADD patients carry single heterozygous variants in *ETFDH* [5, 12–14]. Zhang et al. described a father and his son suffering from late-onset MADD, both of whom carried the same heterozygous null variant in *ETFDH* (c.1285 + 1G > A) [15]. Null variants in *ETFDH* often lead to less residual ETFDH enzyme activity compared to missense variants [7]. In addition, some missense variants cause severe misfolding of variant ETFDH proteins, and some cause milder folding defects [4]. Therefore, we speculate that single heterozygous variants with higher pathogenic impact could cause late-onset MADD, which may be caused by the difference in the compositions of variation types and pathogenicity between late-onset MADD patients carrying biallelic and single heterozygous variations in *ETFDH*.

Therefore, we conducted this meta-analysis to determine the differences in variation types, pathogenicity of missense variants, and clinical characteristics between patients with biallelic and single heterozygous variations in *ETFDH*.

## Methods

We followed the Preferred Reporting Items for Systematic Reviews and Meta-analyses (PRISMA) reporting guideline [16] to perform a systematic review (Supplementary Table 1). The study protocol is available at International Prospective Register of Systematic Reviews (CRD42024529856).

### Eligibility criteria

Studies reporting late-onset MADD patients carrying variations in *ETFDH* gene were included. Case reports, case series of less than five late-onset MADD patients harboring *ETFDH* variations, systematic reviews, conference abstracts, and animal studies were excluded. If patients overlapped between studies, the study with a smaller sample size was excluded. If a patient carried 3 or more variants in *ETFDH*, he/she was excluded from the analysis. Patients with inaccurately characterized variants were excluded from the analysis. The main outcome was the relative frequencies of patients carrying different variation types (null variants and missense variants) between patients with biallelic and single heterozygous variations in *ETFDH* (biallelic group vs heterozygous group). Null variants were defined as nonsense, frameshift, single exon or multiexon deletion, initiation codon, and splice variations, which included canonical ± 1 or 2 splice site variants and intronic variations with a SpliceAI score ≥ 0.5 (https://spliceailookup.broadinstitute.org/). It's worth noting that the classification of null and missense variants is a functional approximation rather than a strict binary categorization. Secondary outcomes included the differences in relative frequencies of hotspot missense variants, computational pathogenicity scores of missense variants, onset ages and serum creatine kinase (CK) levels at diagnosis between the two groups.

### Search methods

A comprehensive search was conducted on December 01, 2024, utilizing six databases: PubMed, Embase, Web of Science, China National Knowledge Infrastructure (CNKI), SinoMed, and Wanfang databases. Although our original protocol registered in PROSPERO specified the use of PubMed, Embase, and Web of Science, we expanded our search strategy to include CNKI, SinoMed, and Wanfang databases. This decision was made to enhance the comprehensiveness of our search and ensure that we captured a broader range of relevant studies, including those published in non-English language journals. Deviations from the pre-registered protocol was that we mented one reference through citation searching to ensure a more comprehensive identification of relevant studies. The detailed search strategies are available in the Supplementary Table 2. The identified studies were then uploaded to Covidence, a web-based software that facilitates citation screening and collaboration among researchers.

### Study selection

Following the elimination of duplicate entries, titles and abstracts were screened independently by two reviewers, H.Q.Z. and J.M. The full texts of potentially relevant articles were then assessed for eligibility basis of on the established inclusion and exclusion criteria, and reasons for excluding studies were systematically documented. Any disagreements were resolved by discussion with a senior author, W.Z., to achieve consensus. The study selection process was clearly illustrated via a PRISMA flow diagram, ensuring transparency and rigor in the review methodology.

### Data collection

Data extraction using a standardized form was conducted independently by two reviewers (H.Q.Z. and J.M.), with disagreements resolved by a third researcher (W.Z.). The following data were collected: name of the first author, year of publication, study design, study period, country, number of patients with late-onset MADD, number of patients with biallelic and single heterozygous variations, variation information, onset ages, serum CK levels at diagnosis, and treatment outcomes for each patient, if reported. References information for all extracted studies can be found in the Supplementary Table 3. The excluded references with less than five late-onset MADD patients harboring *ETFDH* variations were presented in Supplementary Table 4.

### Assessment of bias

Two reviewers H.Q.Z. and J.M. independently used the Joanna Briggs Institute (JBI) Critical Appraisal Checklist for case series to evaluate the risk of bias of each article [17]. The final score of each article was derived from the percentage of affirmative ("yes") responses. Based on these scores, the risk of bias was classified as high (≤ 49%), moderate (50–69%), or low (≥ 70%) [18].

### ACMG classification of variation

The American College of Medical Genetics and Genomics (ACMG) classification of the variations was evaluated according to the Clinical Interpretation of genetic variants by ACMG/AMP 2015 guideline [19] through the WinterVar platform (http://wintervar.wglab.org/).

### Pathogenicity prediction of missense variants

For each detected missense variant in *ETFDH*, its pathogenicity was predicted using sorting intolerant from tolerant (SIFT, http://sift.bii.a-star.edu.sg/) [20], Polymorphism Phenotyping v2 (PolyPhen-2, http://genetics.bwh.harvard.edu/pph2/) [21], Rare Exome Variant Ensemble Learner (REVEL, https://sites.google.com/site/revelgenomics/) [22], Meta-Analytic Support Vector Machine (MetaSVM), Logistic Regression (MetaLR) and Recurrent Neural Network (MetaRNN) (https://sites.google.com/site/jpopgen/dbNSFP) [23, 24]. Summary of pathogenicity prediction tools above were described in the Supplementary Table 5 [20, 21, 23–28].

### Data synthesis and analysis

Statistical analyses were conducted using STATA 18.0 (StataCorp LP, College Station, TX, USA). A DerSimonian and Laird random-effects model meta-analysis with logit transformation was employed to determine the relative frequencies of patients carrying different variation types in patients with late-onset MADD, based on a proportion approach. Subgroup analyses were performed to examine the differences in variation types and the relative frequencies of hotspot missense variations between the biallelic and heterozygous groups. Weighted t-tests utilizing the DerSimonian and Laird random-effects model were conducted to identify the differences in computational pathogenicity scores of missense variants, and patients’ onset ages and serum CK levels at diagnosis between the biallelic and heterozygous groups. The results, including pooled estimates and their 95% confidence intervals (CI), were displayed using forest plots. Heterogeneity among studies was assessed with the *I*^*2*^ statistic and Cochran’s *Q* tests, and a value of *I*^*2*^ > 50% or *P-value (P–H)* < 0.05 for the *Q* test was considered as significant heterogeneity [29]. Because the primary outcome was a meta-analysis of single proportions, publication bias was not assessed in this study [30–32]. Sensitivity analyses for the relative frequency of patients carrying null variants were conducted after excluding studies with a publication date before 2011 and studies only reported patients carrying either biallelic or single heterozygous variations in *ETFDH*. All *P* values were two-sided, and values less than 0.05 were considered statistically significant.

## Results

### Characteristics of included studies

**Fig. 1:**
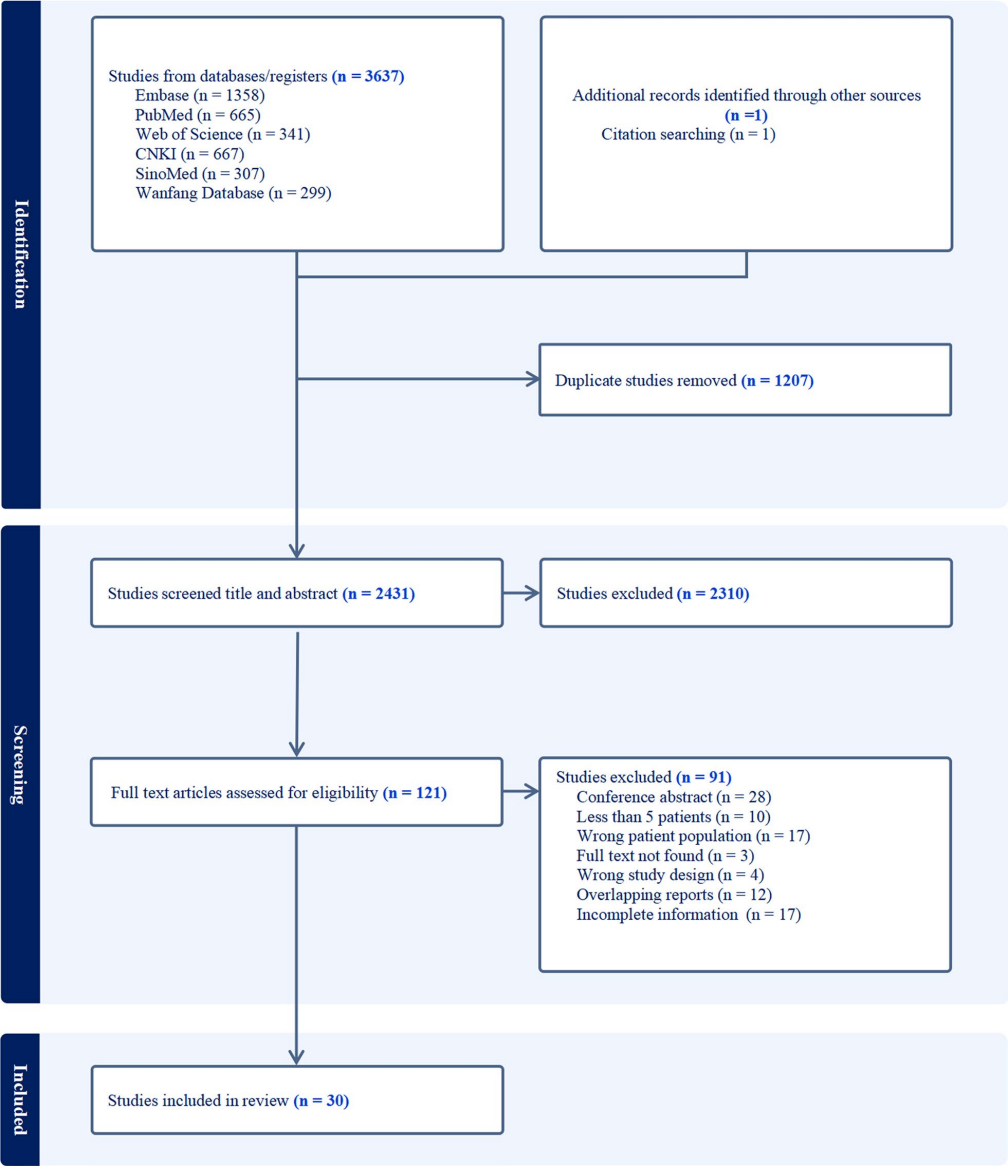
Flow diagram of study selection according to Preferred Reporting Items for Systematic Reviews and Meta-Analyses (PRISMA) guidelines

A search initially identified 3638 studies, from which 1207 duplicates were removed and 2310 were excluded after screening titles and abstracts. Following a full-text review of 121 studies, 91 were further excluded, resulting in 30 eligible studies for inclusion in the meta-analyses with 560 late-onset MADD patients (Fig. 1). All included studies were case series, with their characteristics detailed in Table 1. Most studies were performed in tertiary care centers (27 studies [90.00%]) from 2007 to 2024. The studies were conducted across Asia (23 studies), Europe (6 studies), and South Africa (1 study), with sample sizes ranging from 5 to 106. Among the patients, 498 had biallelic variations and 62 exhibited single heterozygous variations in *ETFDH*.

**Table 1:** Characteristics of the Included Studies

First author	Publication year	Study period	Country	Sample size	Number of patients with biallelic variations	Number of patients with single heterozygous variations
Olsen RK	2007	1998–2006	Denmark	15	14	1
Er TK	2010	NR	China	9	9	0
Lan MY	2010	2001–2008	China	7	7	0
Wang Y	2011	2003–2010	China	23	18	5
Wang ZQ	2011	NR	China	53	52	1
Xi JY	2011	2005–2010	China	30	28	2
Zhu M	2014	2011–2013	China	12	7	5
Béhin A	2016	NR	France	13	11	2
Liu XY	2016	2012–2015	China	28	23	5
Angelini C	2018	NR	Italy	6	6	0
Zhao YW	2018	2013–2016	China	21	14	7
Hong DJ	2019	2014–2018	China	25	17	8
Nilipour Y	2020	2011–2016	Iran	19	19	0
Sun YM	2020	2012–2017	China	5	4	1
Yildiz Y	2020	NR	Turkey	20	20	0
Yuan J	2020	2009–2019	China	14	9	5
Ali A	2021	2009–2020	United Arab Emirates	10	10	0
Kuo YC	2021	1998–2018	China	5	5	0
Staretz-Chacham O	2021	NR	Israel	5	5	0
Tang Z	2021	2018–2021	China	5	5	0
Liu HY	2022	2009–2021	China	26	24	2
Lupica A	2022	2001–2021	Italy	10	7	3
Wen B	2022	1995–2019	China	106	101	5
Yamada K	2022	1997–2020	Japan	17	15	2
Zhang J	2022	2005–2020	China	25	18	7
Zheng W	2022	2019–2021	China	12	12	0
Zhang HQ	2023	2016–2022	China	11	9	2
Bilgin H	2024	2017–2022	Turkey	6	6	0
Bisschof M	2024	2021–2023	South Africa	10	10	0
Schee JP	2024	1994–2024	Malaysia	13	13	0

*NR* not reported

### Risk of bias of included studies

The evaluation of bias risk, conducted with the JBI checklists, indicated that all included studies were categorized as low risk (Supplementary Table 6).

### Variation types in patients with late-onset MADD

**Fig. 2:**
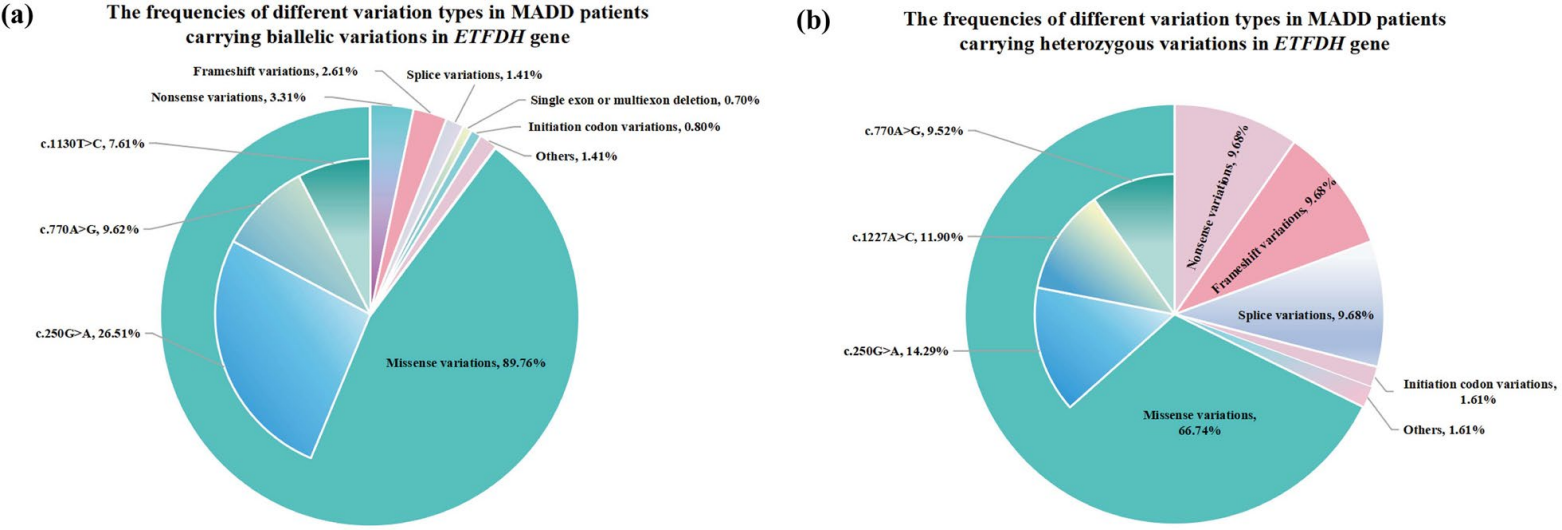
Pie chart representing the frequencies of the different variation types in patients carrying biallelic (**a**) and single heterozygous variations (**b**) in *ETFDH* gene

Thirty and seventeen studies reported late-onset patients carrying biallelic and single heterozygous *ETFDH* variations, respectively. The pie charts indicated the relative proportions of different variation types in patients carrying *ETFDH* variations (Fig. 2). In the biallelic group, the missense variations presented the highest proportion (89.76%), followed by nonsense variations (3.31%). The missense variations also presented the highest proportion (66.74%) in the single heterozygous group, followed by nonsense (9.68%), frameshift (9.68%) and splice (9.68%) variations.

**Fig. 3:**
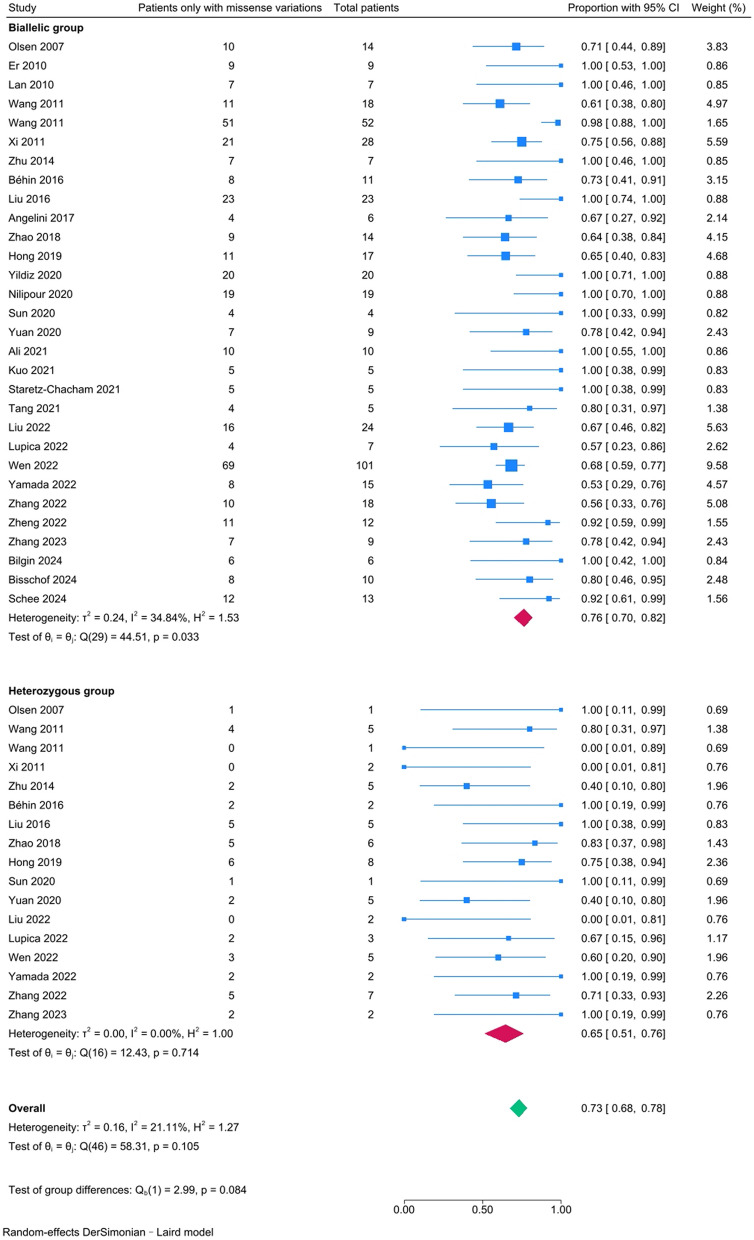
The forest plot for the proportions of patients only carrying missense variations in the biallelic and heterozygous groups

The relative frequency of patients only carrying missense variations in *ETFDH* was 73% (95% CI [68–78%]). There was a low degree of heterogeneity across studies (*I*^*2*^ = 21.11%; *P–H* = 0.105). Subgroup analyses revealed that the relative frequency was 76% (95% CI [70%-82%], *I*^*2*^ = 34.84%; *P–H* = 0.033) in the biallelic group, which was higher than that in the heterozygous group (65%, 95% CI [51%-76%], *I*^*2*^ = 0.00%; *P–H* = 0.714), although the difference did not reach statistical significance (*P* = 0.084) (Fig. 3).

**Fig. 4:**
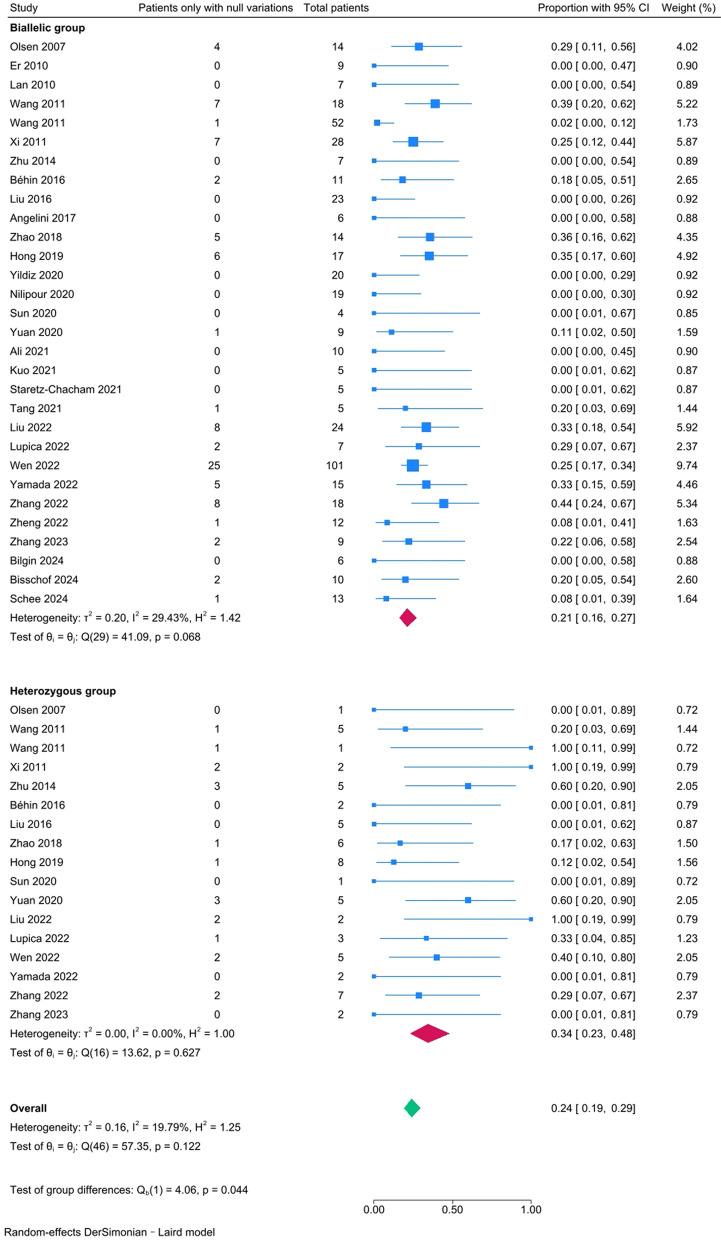
The forest plot for the proportions of patients carrying null variations in the biallelic and heterozygous groups

The relative frequency of patients carrying null variants in *ETFDH* was 24% (95% CI [19%-29%]). There was no heterogeneity among studies (*I*^*2*^ = 19.79%, *P–H* = 0.122). In subgroup analyses, the percentage in the biallelic group (21%, 95% CI [16%-27%], *I*^*2*^ = 29.43%, *P–H* = 0.068) was significantly lower than that in the heterozygous group (34%, 95% CI [23%-48%], *I*^*2*^ = 0.00%, *P–H* = 0.627; *P* = 0.044) (Fig. 4). And the results were similar after excluding patients carrying variants of uncertain significance (VUS) classified by ACMG classification (*P* = 0.008) (Supplementary Fig. 1). The sensitivity analyses did not show any substantial variation after excluding studies with a publication date before 2011 and studies only reporting patients carrying either biallelic or single heterozygous variations in *ETFDH* (Table 2).

**Table 2:** Sensitivity analyses after excluding studies with a publication date before 2011 and only reporting patients carrying either biallelic or single heterozygous variations in *ETFDH*

Characteristic	Number of studies combined	Total number of patients	Proportion of patients with null variations (95% CI)	*I*^*2*^, %	*P* value*
Years of publication (2011–2024)			0.24 (0.19, 0.30)	22.73	0.047
Biallelic variations	27	468	0.21 (0.16, 0.28)	32.05	
Single heterozygous variations	16	61	0.35 (0.231, 0.488)	0.00	
Genotype (Biallelic and single heterozygous variations)			0.25 (0.19., 0.31)	29.38	0.048
Biallelic variations	23	424	0.21 (0.15, 0.28)	37.86	
Single heterozygous variations	14	56	0.36 (0.23, 0.51)	0.87	

^*^represents the *P* value between the groups of biallelic variations and single heterozygous variations

### Relative frequencies of hotspot missense variations in *ETFDH* in the two groups

The three most common missense variations in *ETFDH* are c.250G > A (A84T, 237/894), c.770A > G (Y257C, 86/894), and c.1130T > C (L337P, 68/894) in late-onset MADD patients with biallelic variations, and c.250G > A (A84T, 6/42), c.1227A > C (L409F, 5/42), and c.770A > G (Y257C, 4/42) in those with single heterozygous variations (Fig. 2). Of the four variants mentioned above, the relative frequencies of c.1227A > C (8% vs. 19%, *P* = 0.006) and c.1130T > C (4% vs 12%, *P* = 0.049) were different between the two groups (Supplementary Figs. 2, 3, 4, 5).

### Pathogenicity scores of missense variations in the two groups

Compared with the heterozygous group, the biallelic group presented significantly higher SIFT scores (*P* < 0.001), lower PolyPhen-2 scores (*P* < 0.001), and lower MetaRNN scores (*P* < 0.001), indicating less pathogenicity than the heterozygous group (Supplementary Figs. 6, 7 and 8). However, there were no significant differences between the groups in the scores of REVEL (*P* = 0.954), MetaSVM (*P* = 0.193) and MetaLR (*P* = 0.445) (Supplementary Figs. 9, 10 and 11). Ensemble predictors (REVEL, MetaSVM, MetaLR) did not support a significant difference, which weakened the strength for the stronger pathogenicity of missense variations in the heterozygous group.

### Onset ages, serum CK levels at diagnosis and treatment outcomes in the two groups

A total of 23 studies reported the onset ages and 17 reported serum CK levels in patients with biallelic variations, while 9 and 8 studies reported these measures in patients with single heterozygous variations, respectively. Subgroup analyses revealed that patients with biallelic variations exhibited a younger onset age (*P* < 0.001) and a higher serum CK level (*P* = 0.002), indicating a severer phenotype (Supplementary Figs. 12, 13). In addition, twenty-five studies reported the treatment outcomes for a total of 404 patients, of whom 97.03% (392/404) showed clinical improvement after riboflavin treatment.

## Discussion

To our knowledge, this is the first meta-analysis to document the relative frequencies of variation types in patients with late-onset MADD. Patients with single heterozygous variations in *ETFDH* exhibited a higher proportion of null variations compared to those with biallelic variations. The results demonstrated significant disparities in variation types across the groups.

It is curious why some late-onset MADD patients carry single heterozygous variants in *ETFDH* in the context of that the disease is widely regarded as an autosomal recessive disease. A hypothesis has been proposed by several researchers: there is a second variant in regulatory regions or in intronic splicing regulatory elements which is undetected by sequencing assays [12, 13]. If entirely true, the compositions of variation types should be the same between the biallelic and heterozygous groups. However, our study revealed that the heterozygous group was more likely to carry null variants than the biallelic group. Null variants in *ETFDH* often lead to less residual ETFDH enzyme activity than missense variants [7]. As demonstrated in *KMT2B*-related disorders, patients with null variants also exhibited a higher disease burden than those with missense variations [33]. These findings indicate that the relationship between* ETFDH* variant types and the onset of MADD is complex. Our analysis reveals exploratory differences in pathogenicity predictions between the single heterozygous and biallelic groups, although these findings are not consistent across all prediction tools. Future research should focus on larger cohorts with detailed clinical data to better understand the relationship between variant type and disease onset in MADD.

We acknowledge that the studies included predominantly utilized WES for detection, which may lead to the omission of the second variants (e.g., deep intronic or structural variants). Additionally, the role of genetic modifiers cannot be overlooked. However, we would like to particularly emphasize the situation is complex as the disease onset is influenced by environmental factors. We hypothesize that a possible underlying mechanism is gene-environment interactions, that is the residual ETFDH enzyme activity caused by the single heterozygous variations in *ETFDH* with stronger pathogenicity would be further reduced below the threshold under the influence of environmental factors, ultimately leading to the manifestation of the MADD phenotype. Most patients with late-onset MADD developed symptoms or deteriorated upon infection or metabolic stress. In particular, riboflavin deficiency contributes greatly to the pathogenesis of late-onset MADD [34]. FAD, an active form of riboflavin, promotes folding and conformational stabilization of ETFDH [4], and riboflavin deficiency decreases the amount of ETFDH protein in fibroblasts from late-onset MADD patients and healthy subjects. Most late-onset MADD patients have decreased serum riboflavin and muscular FAD levels [34, 35], and responded well to riboflavin treatment. In the largest Chinese cohort of late-onset MADD, 13 of 48 patients took no further riboflavin administration till the endpoint of follow-up after an average of 2.2 months riboflavin treatment, and kept clinically cured status (the longest follow-up time was 13 years) [34].

Regarding clinical severity, our study revealed a younger onset age and a higher serum CK level in the biallelic group compared to the heterozygous group, suggesting milder clinical manifestations in heterozygous carriers. One potential explanation for these observations is the biallelic group may be attributed to the cumulative effect of biallelic variations on enzyme function, leading to more pronounced metabolic disturbances and muscle involvement. However, further functional studies are necessary to validate this hypothesis and elucidate the precise mechanisms involved in disease manifestation.

Therefore, it is crucial to pay close attention to individuals carrying single heterozygous variants in *ETFDH* gene in clinical practice, early clinical intervention and guidance should be provided to prevent the occurrence of disease progression and metabolic crises. This awareness can lead to more accurate and timely diagnoses, enabling early intervention and management. In terms of screening, it suggests that current genetic screening strategies may need to be expanded, potentially incorporating functional assays or additional genetic analyses to identify at-risk individuals more effectively.

Limitations of our study included the following: (1) exclusion criteria that omitted studies with overlapping participants and small sample sizes, (2) potential selection bias from hospital-based case series, and (3) a limited number of patients with single heterozygous variants due to the rarity and inheritance mode of the disease, (4) the exclusion of unpublished or grey literature, (5) insufficient information of treatment outcomes, thus providing limited guidance on clinical prognosis, (6) incomplete information of deep intronic variants detection across included studies, and (7) no further analysis of stratifying the clinical data by variant type due to data limitations.

In conclusion, our study demonstrates that the compositions of variation types differ between late-onset MADD patients with biallelic and single heterozygous variations, with a higher proportion of null variants in patients with heterozygous variations. Our study highlights the importance of re-evaluating the inheritance pattern and pathogenic mechanisms in late-onset MADD. In clinical practice, further research is needed to confirm the role of single heterozygous variants and to develop targeted interventions for patients at risk.

## Supplementary Information

Supplementary file 1. Fig. 1 The forest plot for the proportions of patients carrying null variations after omitting VUS variants in the biallelic and heterozygous groups.

Supplementary file 2 . Fig. 2 The forest plot for the relative frequencies of c.250G>A in patients carrying biallelic and single heterozygous variations in *ETFDH* gene.

Supplementary file 3. Fig. 3 The forest plot for the relative frequencies of c.770A>G in patients carrying biallelic and single heterozygous variations in *ETFDH* gene.

Supplementary file 4. Fig. 4 The forest plot for the relative frequencies of c.1130T>C in patients carrying biallelic and single heterozygous variations in *ETFDH* gene.

Supplementary file 5. Fig. 5 The forest plot for the relative frequencies of c.1227A>C in patients carrying biallelic and single heterozygous variations in *ETFDH* gene.

Supplementary file 6. Fig. 6 The forest plot for the SIFT scores in patients carrying biallelic and single heterozygous variations in *ETFDH* gene.

Supplementary file 7. Fig. 7. The forest plots for the PolyPhen-2 scores in patients carrying biallelic and single heterozygous variations in *ETFDH* gene.

Supplementary file 8. Fig. 8 The forest plot for the MetaRNN scores in patients carrying biallelic and single heterozygous variations in *ETFDH* gene.

Supplementary file 9. Fig. 9 The forest plot for the REVEL scores in patients carrying biallelic and single heterozygous variations in *ETFDH* gene.

Supplementary file 10. Fig. 10 The forest plot for the MetaSVM scores in patients carrying biallelic and single heterozygous variations in *ETFDH* gene.

Supplementary file 11. Fig. 11 The forest plot for the MetaLR scores in patients carrying biallelic and single heterozygous variations in *ETFDH* gene.

Supplementary file 12. Fig. 12 The forest plot for weighted means of onset ages in patients carrying biallelic and single heterozygous variations in *ETFDH* gene.

Supplementary file 13. Fig. 13 The forest plot for weighted means of serum CK levels in patients carrying biallelic and single heterozygous variations in *ETFDH* gene.

Supplementary file 14.

Supplementary file 15.

Supplementary file 16.

Supplementary file 17.

Supplementary file 18.

Supplementary file 19.

## Data Availability

Data supporting the findings of this study are available within the paper and its Supplementary Information.
