# Supplementary figures and images for: The difference of variation types between late-onset multiple acyl-CoA dehydrogenase deficiency patients carrying biallelic and single heterozygous variations in ETFDH: a systematic review and meta-analysis
Source: Orphanet J Rare Dis. 2025 Jun 18;20:310. doi: 10.1186/s13023-025-03845-7 (PMC12178022; doi:10.1186/s13023-025-03845-7)

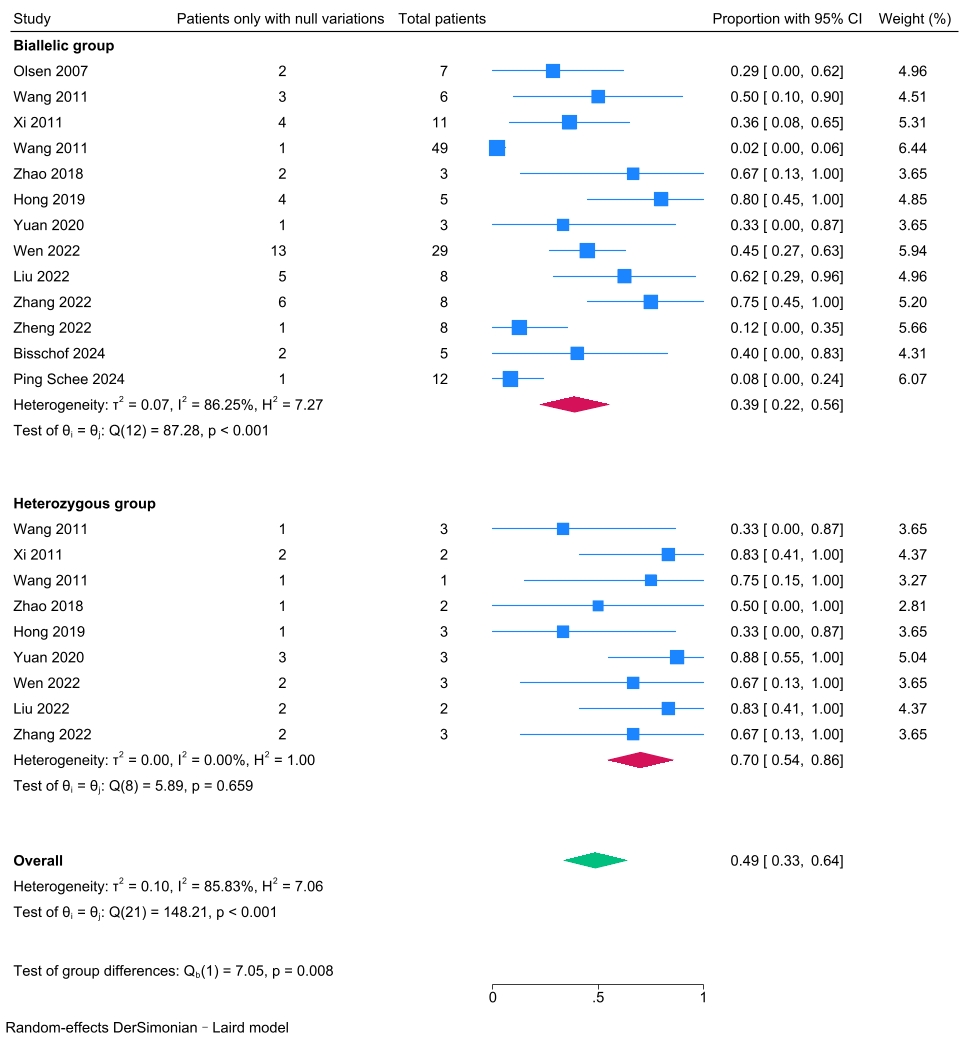

Supplement: Supplementary file 1 — Fig. 1 The forest plot for the proportions of patients carrying null variations after omitting VUS variants in the biallelic and heterozygous groups. [file 13023_2025_3845_MOESM1_ESM.jpg]

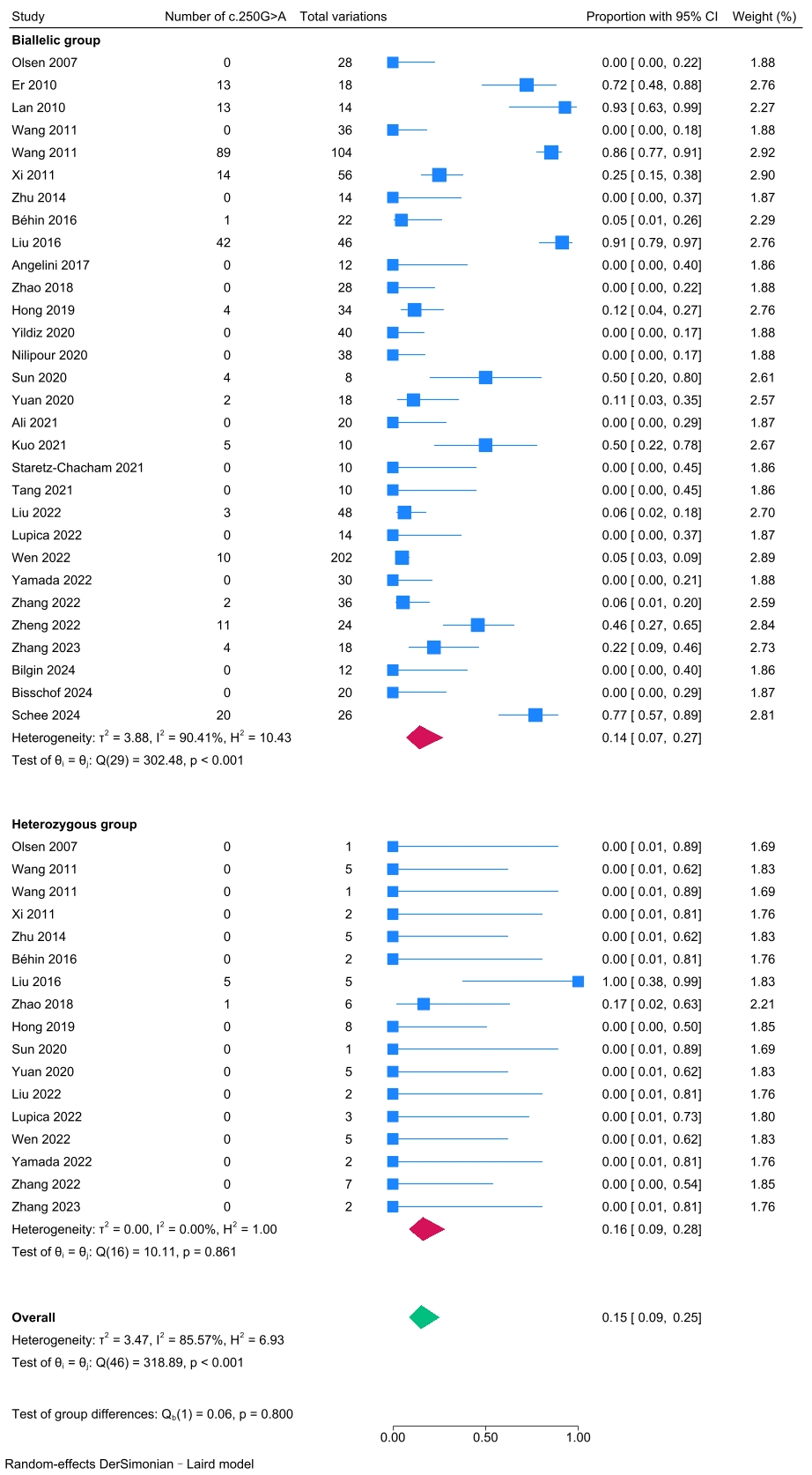

Supplement: Supplementary file 2 — Fig. 2 The forest plot for the relative frequencies of c.250G>A in patients carrying biallelic and single heterozygous variations in ETFDH gene. [file 13023_2025_3845_MOESM2_ESM.jpg]

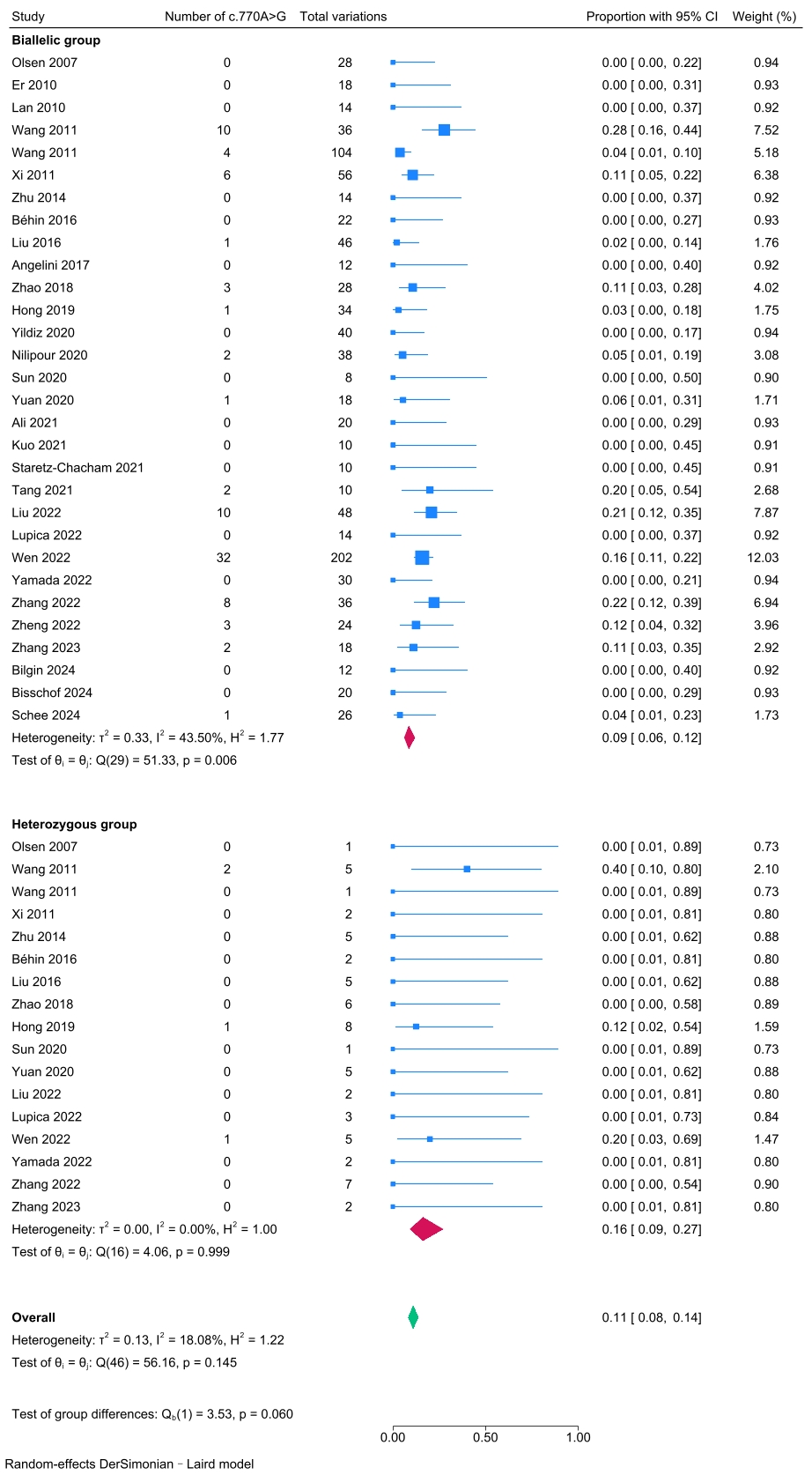

Supplement: Supplementary file 3 — Fig. 3 The forest plot for the relative frequencies of c.770A>G in patients carrying biallelic and single heterozygous variations in ETFDH gene. [file 13023_2025_3845_MOESM3_ESM.jpg]

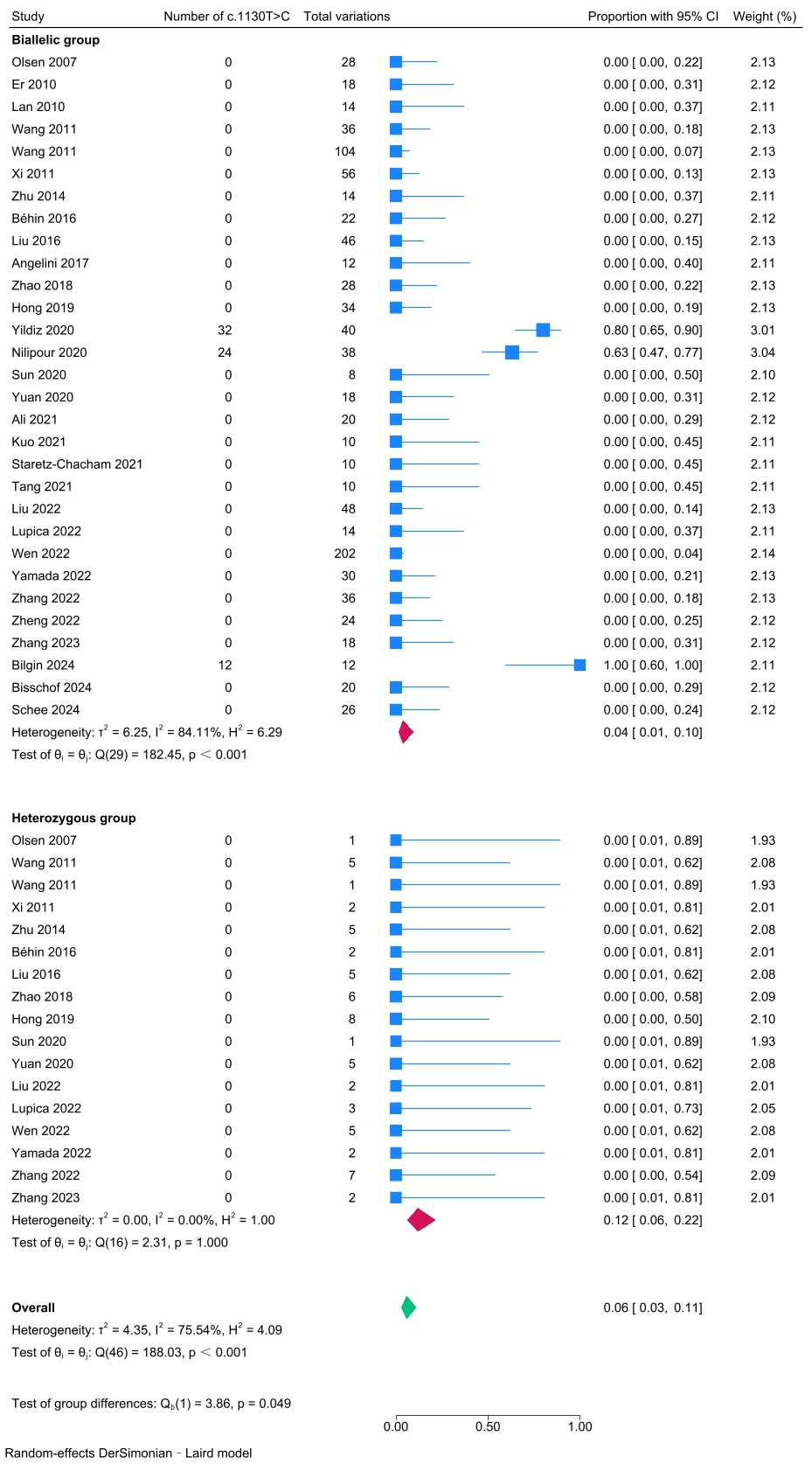

Supplement: Supplementary file 4 — Fig. 4 The forest plot for the relative frequencies of c.1130T>C in patients carrying biallelic and single heterozygous variations in ETFDH gene. [file 13023_2025_3845_MOESM4_ESM.jpg]

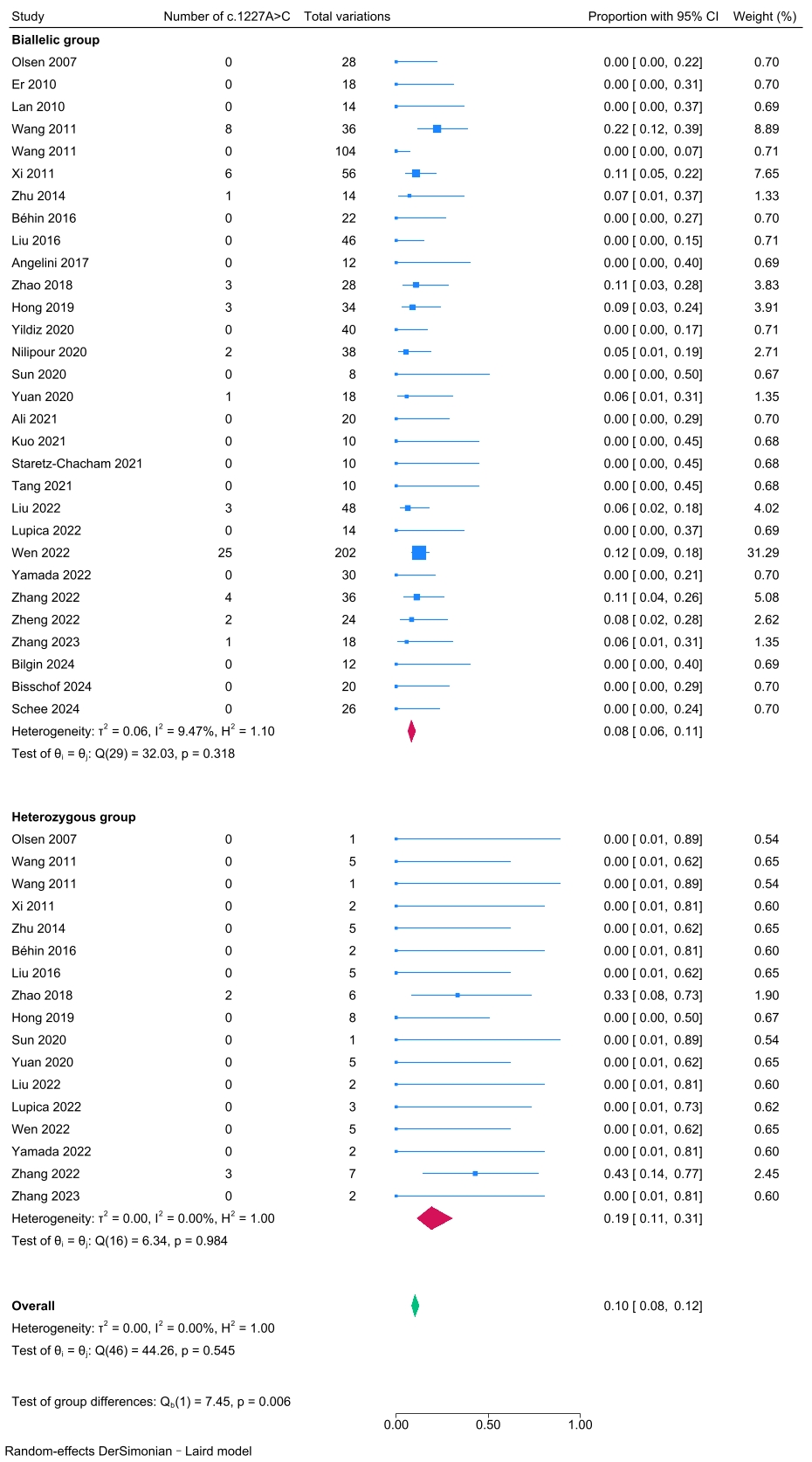

Supplement: Supplementary file 5 — Fig. 5 The forest plot for the relative frequencies of c.1227A>C in patients carrying biallelic and single heterozygous variations in ETFDH gene. [file 13023_2025_3845_MOESM5_ESM.jpg]

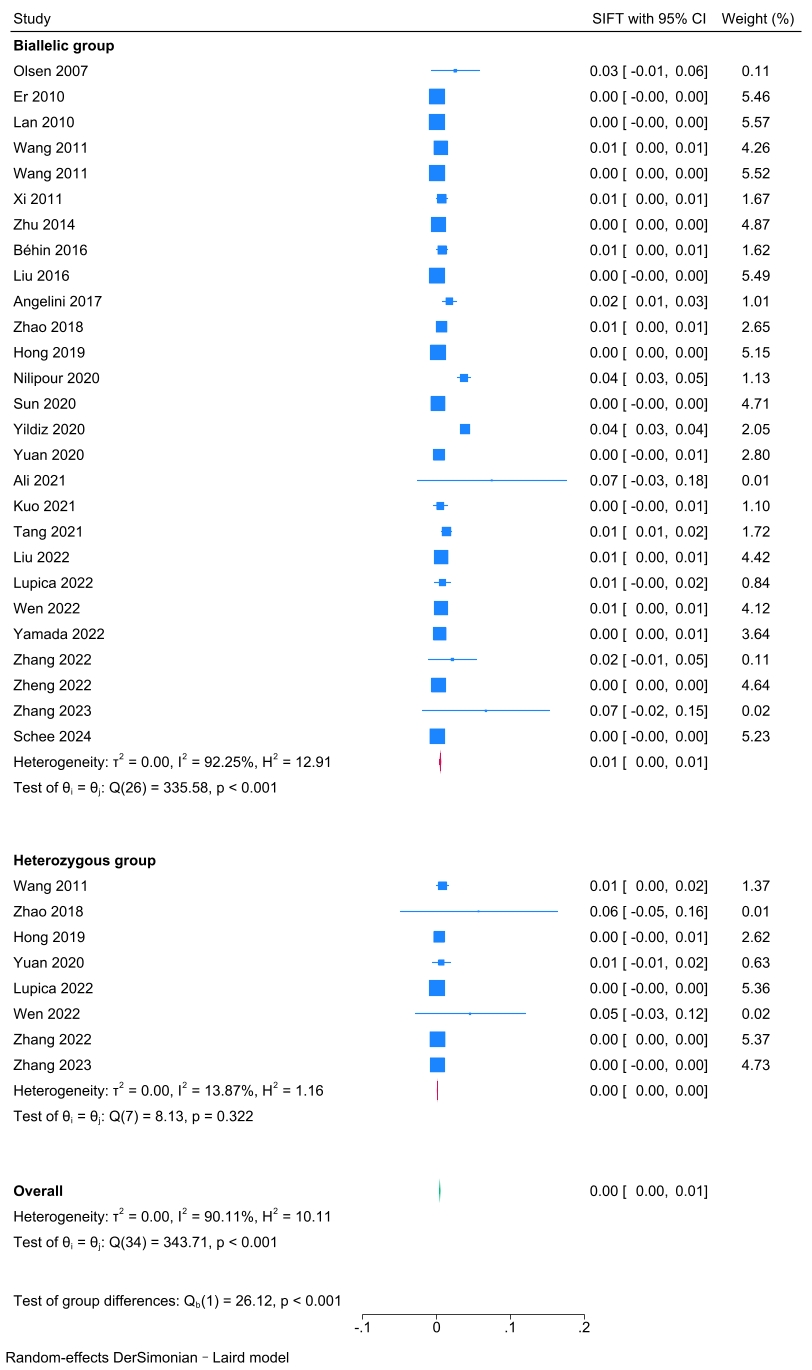

Supplement: Supplementary file 6 — Fig. 6 The forest plot for the SIFT scores in patients carrying biallelic and single heterozygous variations in ETFDH gene. [file 13023_2025_3845_MOESM6_ESM.jpg]

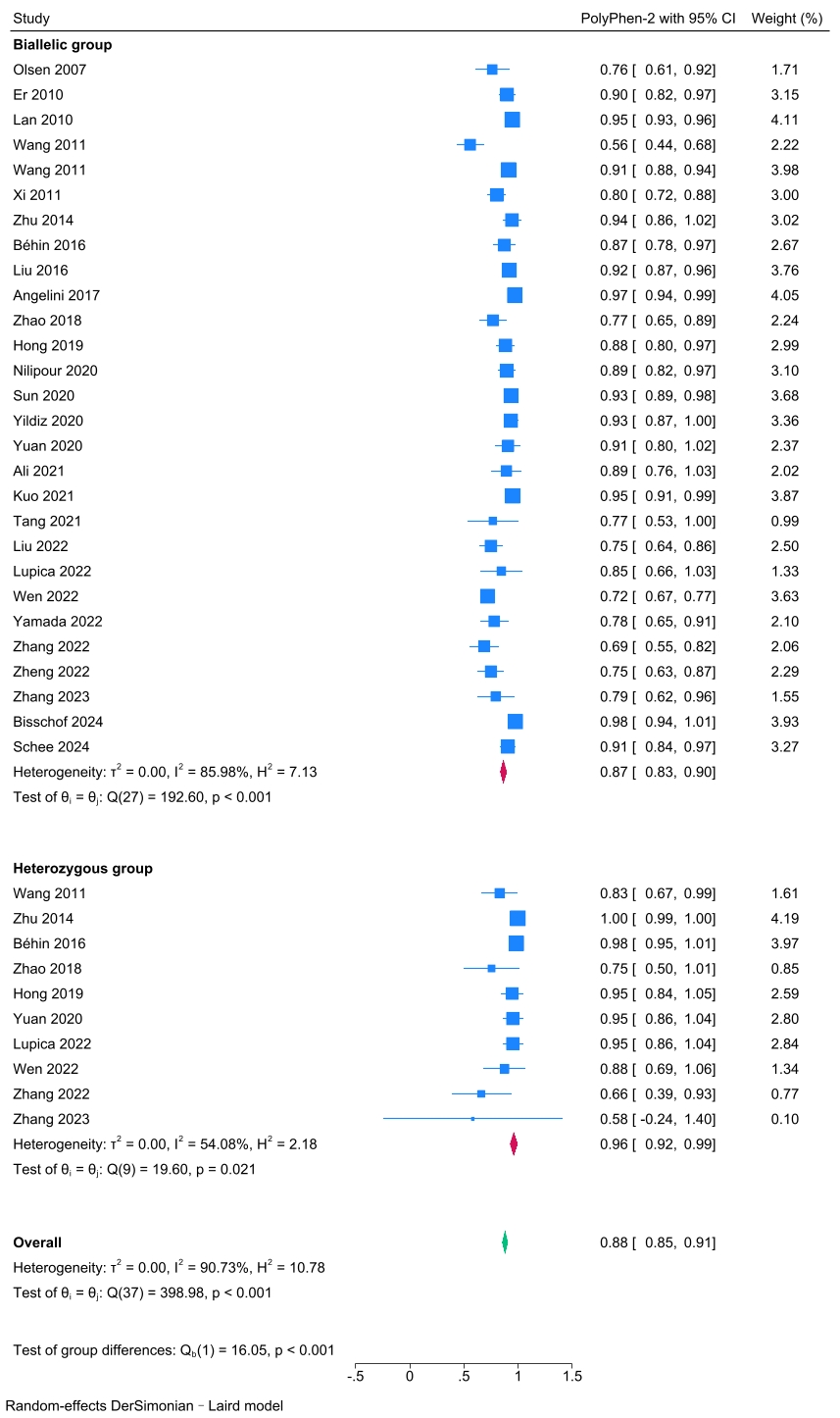

Supplement: Supplementary file 7 — Fig. 7. The forest plots for the PolyPhen-2 scores in patients carrying biallelic and single heterozygous variations in ETFDH gene. [file 13023_2025_3845_MOESM7_ESM.jpg]

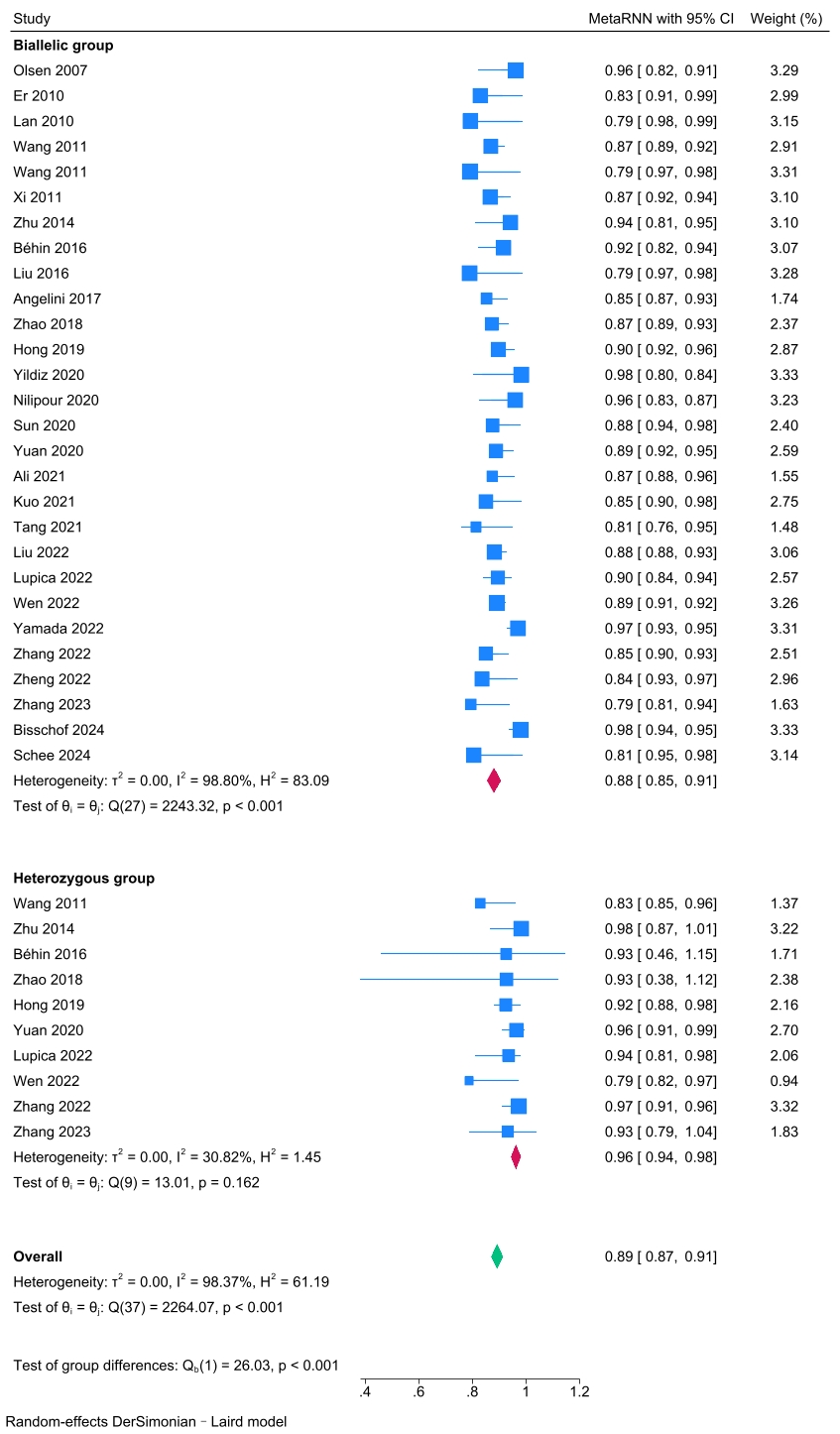

Supplement: Supplementary file 8 — Fig. 8 The forest plot for the MetaRNN scores in patients carrying biallelic and single heterozygous variations in ETFDH gene. [file 13023_2025_3845_MOESM8_ESM.jpg]

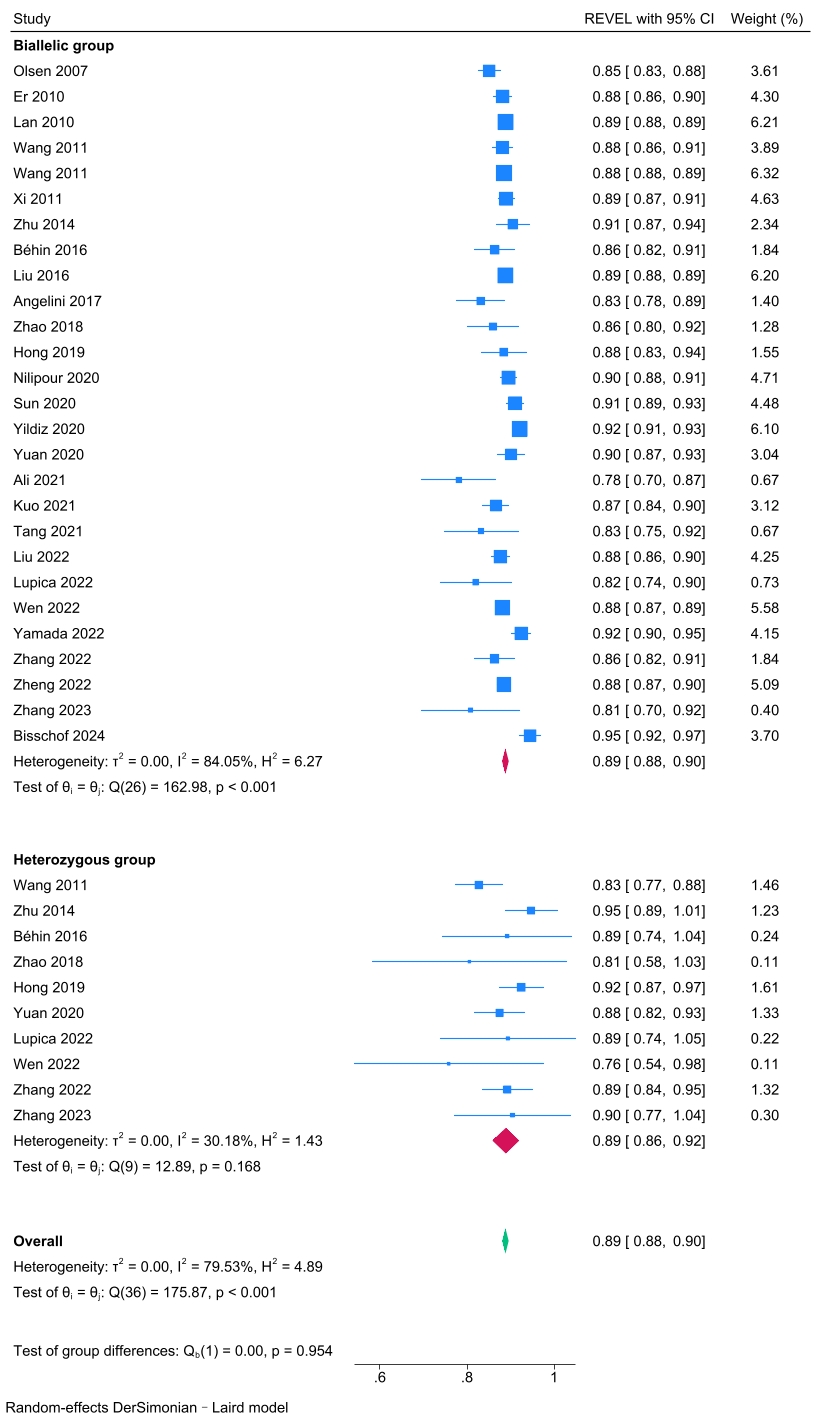

Supplement: Supplementary file 9 — Fig. 9 The forest plot for the REVEL scores in patients carrying biallelic and single heterozygous variations in ETFDH gene. [file 13023_2025_3845_MOESM9_ESM.jpg]

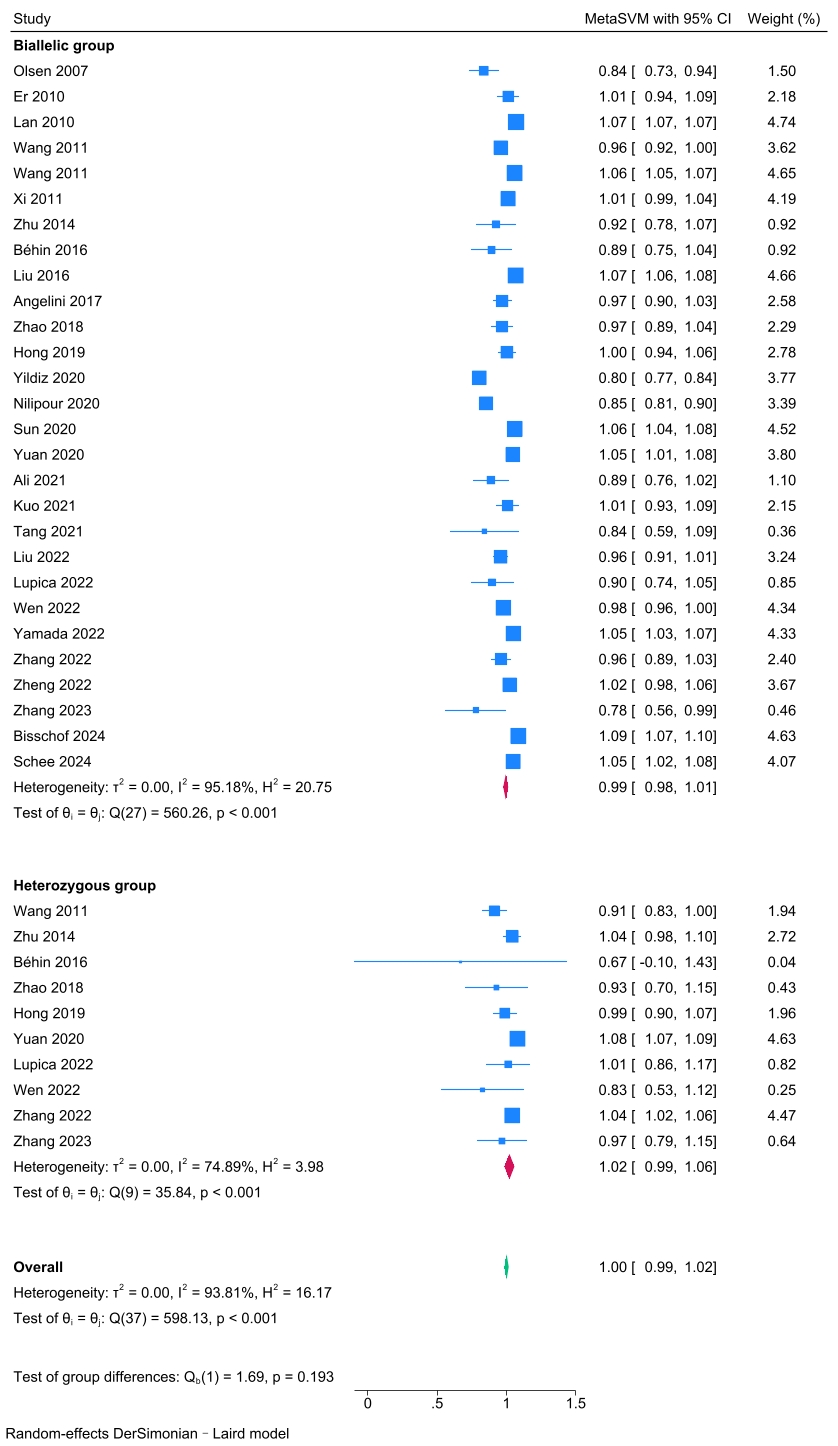

Supplement: Supplementary file 10 — Fig. 10 The forest plot for the MetaSVM scores in patients carrying biallelic and single heterozygous variations in ETFDH gene. [file 13023_2025_3845_MOESM10_ESM.jpg]

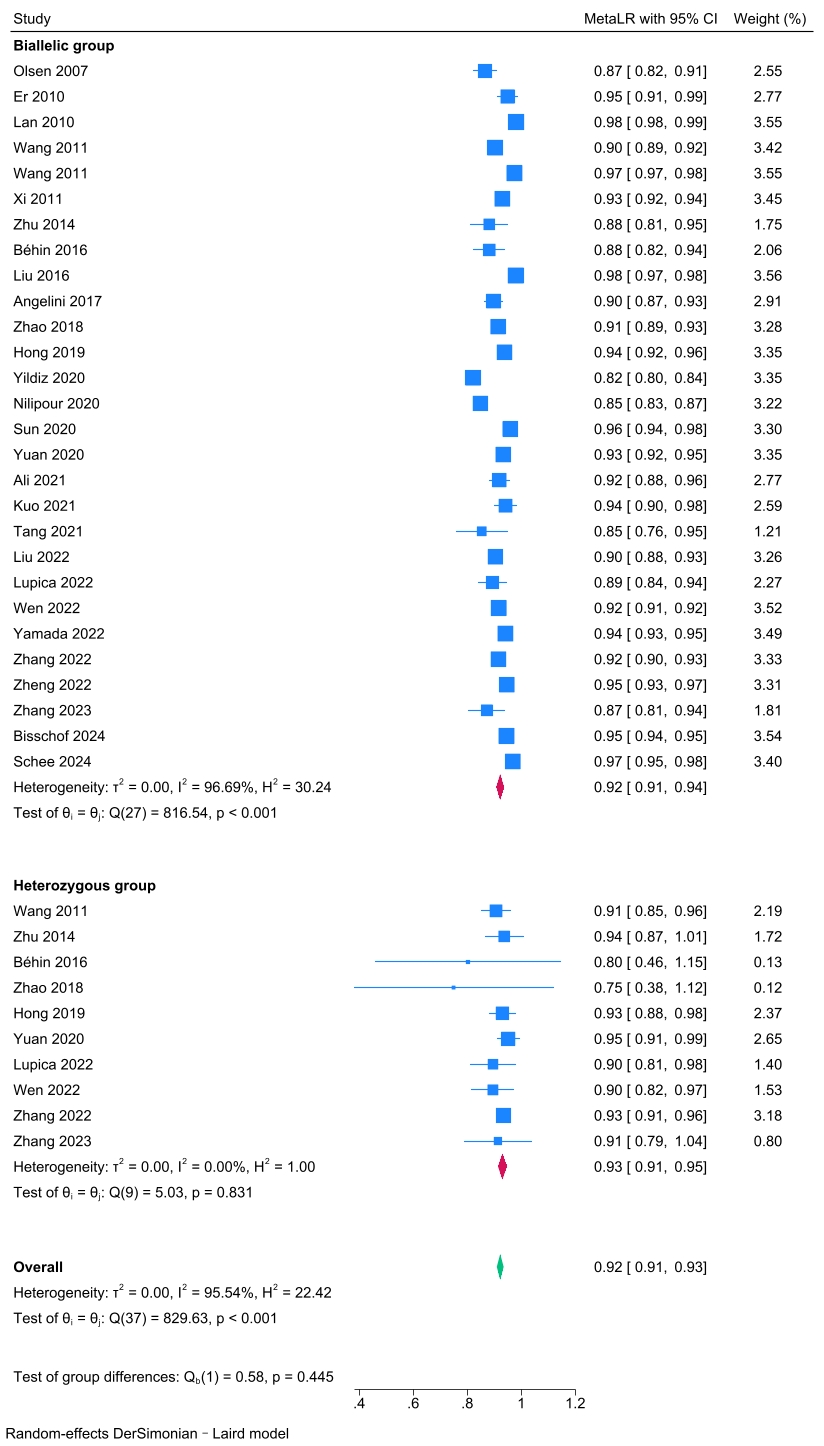

Supplement: Supplementary file 11 — Fig. 11 The forest plot for the MetaLR scores in patients carrying biallelic and single heterozygous variations in ETFDH gene. [file 13023_2025_3845_MOESM11_ESM.jpg]

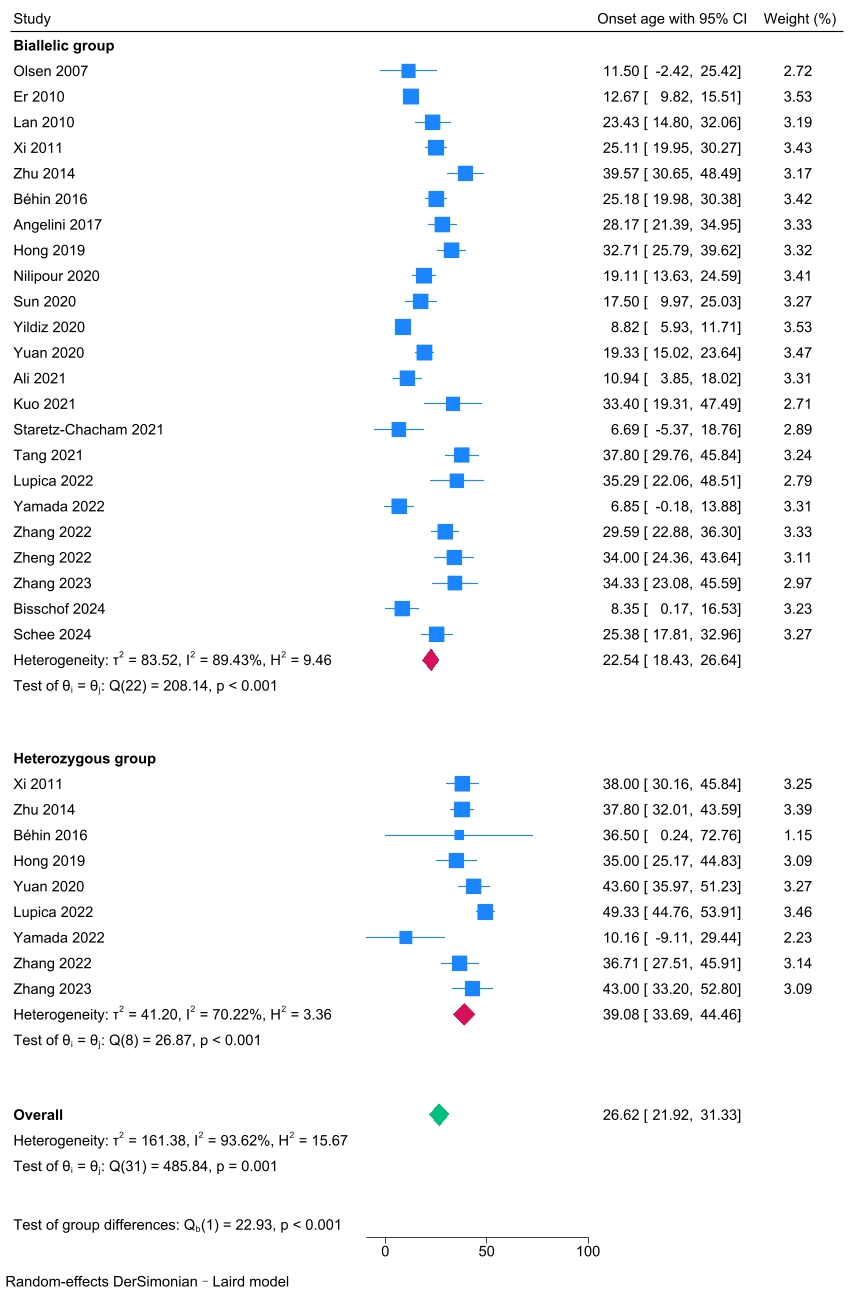

Supplement: Supplementary file 12 — Fig. 12 The forest plot for weighted means of onset ages in patients carrying biallelic and single heterozygous variations in ETFDH gene. [file 13023_2025_3845_MOESM12_ESM.jpg]

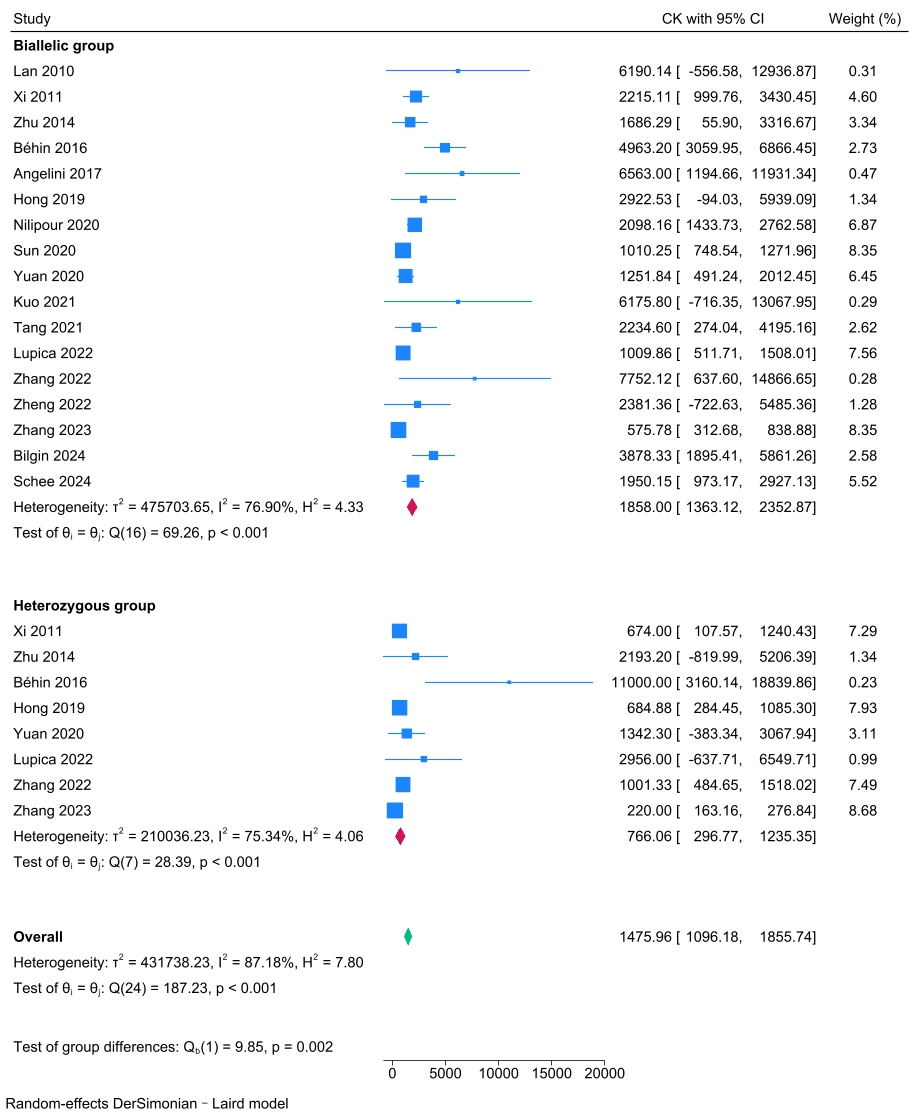

Supplement: Supplementary file 13 — Fig. 13 The forest plot for weighted means of serum CK levels in patients carrying biallelic and single heterozygous variations in ETFDH gene. [file 13023_2025_3845_MOESM13_ESM.jpg]
