# Supplementary material for: The difference of variation types between late-onset multiple acyl-CoA dehydrogenase deficiency patients carrying biallelic and single heterozygous variations in ETFDH: a systematic review and meta-analysis
Source: Orphanet J Rare Dis. 2025 Jun 18;20:310. doi: 10.1186/s13023-025-03845-7 (PMC12178022; doi:10.1186/s13023-025-03845-7)
Supplement: Supplementary file 15 [file 13023_2025_3845_MOESM15_ESM.docx]

| **Database Searched** | **Search Words** | **Search Strategy** | **Results** |
| --- | --- | --- | --- |
| PubMed | “multiple acyl-CoA dehydrogenase deficiency” or “glutaric aciduria II” or “glutaric acidemia II” | "multiple acyl coenzyme a dehydrogenase deficiency"[MeSH Terms] OR "multiple acyl coenzyme a dehydrogenase deficiency"[All Fields] OR ("glutaric"[All Fields] AND "acidemia"[All Fields] AND "ii"[All Fields]) OR "glutaric acidemia ii"[All Fields] OR ("multiple acyl coenzyme a dehydrogenase deficiency"[MeSH Terms] OR "multiple acyl coenzyme a dehydrogenase deficiency"[All Fields] OR ("glutaric"[All Fields] AND "aciduria"[All Fields] AND "ii"[All Fields]) OR "glutaric aciduria ii"[All Fields]) OR ("multiple acyl coenzyme a dehydrogenase deficiency"[MeSH Terms] OR "multiple acyl coenzyme a dehydrogenase deficiency"[All Fields] OR ("multiple"[All Fields] AND "acyl"[All Fields] AND "coa"[All Fields] AND "dehydrogenase"[All Fields] AND "deficiency"[All Fields]) OR "multiple acyl coa dehydrogenase deficiency"[All Fields]) | 665 |
| Embase | “multiple acyl-CoA dehydrogenase deficiency” or “glutaric aciduria II” or “glutaric acidemia II” | 'multiple acyl-coa dehydrogenase deficiency'/exp OR 'multiple acyl-coa dehydrogenase deficiency' OR (multiple AND ('acyl coa'/exp OR 'acyl coa') AND ('dehydrogenase'/exp OR dehydrogenase) AND ('deficiency'/exp OR deficiency)) OR 'glutaric aciduria II' OR (glutaric AND ('aciduria'/exp OR aciduria) AND II) OR 'glutaric acidemia II' OR (glutaric AND ('acidemia'/exp OR acidemia) AND II) | 1358 |
| Web of Science | “multiple acyl-CoA dehydrogenase deficiency” or “glutaric aciduria II” or “glutaric acidemia II” | ((ALL=(multiple acyl-CoA dehydrogenase deficiency)) OR ALL=(glutaric aciduria II)) OR ALL=(glutaric acidemia II) | 341 |
| CNKI | “multiple acyl-CoA dehydrogenase deficiency” or “glutaric aciduria II” or “glutaric acidemia II” | 全部:(多酰基辅酶A脱氢酶缺乏症) or 全部:(多酰基辅酶A脱氢酶缺乏) or 全部:(多酰基辅酶脱氢酶缺乏症) or 全部:(多酰基辅酶脱氢酶缺乏) or 全部:(戊二酸血症) or 全部:(戊二酸尿症) | 667 |
| SinoMed | “multiple acyl-CoA dehydrogenase deficiency” or “glutaric aciduria II” or “glutaric acidemia II” | "多酰基辅酶A脱氢酶缺乏症"[全部字段:智能] OR "多酰基辅酶A脱氢酶缺乏"[全部字段:智能] OR "多酰基辅酶脱氢酶缺乏症"[全部字段:智能] OR "多酰基辅酶脱氢酶缺乏"[全部字段:智能] OR "戊二酸尿症"[全部字段:智能] OR "戊二酸血症"[全部字段:智能] | 307 |
| WanFangData | “multiple acyl-CoA dehydrogenase deficiency” or “glutaric aciduria II” or “glutaric acidemia II” | (全文=多酰基辅酶A脱氢酶缺乏症) OR (全文=多酰基辅酶A脱氢酶缺乏) OR (全文=多酰基辅酶脱氢酶缺乏症) OR (全文=多酰基辅酶脱氢酶缺乏) OR (全文=戊二酸血症)OR (全文=戊二酸尿症) | 299 |
| Total |  |  | **3637** |

**Supplemental Table 2** The detailed search strategies and results.
