# Supplementary material for: The difference of variation types between late-onset multiple acyl-CoA dehydrogenase deficiency patients carrying biallelic and single heterozygous variations in ETFDH: a systematic review and meta-analysis
Source: Orphanet J Rare Dis. 2025 Jun 18;20:310. doi: 10.1186/s13023-025-03845-7 (PMC12178022; doi:10.1186/s13023-025-03845-7)
Supplement: Supplementary file 16 [file 13023_2025_3845_MOESM16_ESM.docx]

**Supplementary Table 3** References information for all extracted studies.

| First author | Publication year | Title | DOI |
| --- | --- | --- | --- |
| Olsen RK | 2007 | ETFDH mutations as a major cause of riboflavin-responsive multiple acyl-CoA dehydrogenation deficiency | 10.1093/brain/awm135 |
| Er TK | 2010 | High resolution melting analysis facilitates mutation screening of ETFDH gene: Applications in riboflavin-responsive multiple acyl-CoA dehydrogenase deficiency | 10.1016/j.cca.2010.01.033 |
| Lan MY | 2010 | High frequency of ETFDH c.250G>A mutation in Taiwanese patients with late-onset lipid storage myopathy | 10.1111/j.1399-0004.2010.01421.x |
| Wang Y | 2011 | Hot spot mutations in electron transfer flavoprotein dehydrogenase gene of riboflavin responsive lipid storage myopathy in 20 Chinese families | 10.3760/cma.j.issn.1006-7876.2011.05.005 |
| Wang ZQ | 2011 | Molecular analysis of 51 unrelated pedigrees with late-onset multiple acyl-CoA dehydrogenation deficiency (MADD) in southern China confirmed the most common ETFDH mutation and high carrier frequency of c.250G>A | 10.1007/s00109-011-0725-7 |
| Xi JY | 2011 | Clinical features and electron transfer flavoprotein dehydrogenase gene mutation analysis in 35 Chinese patients with lipid storage myopathy | 10.1007/s00109-011-0725-7 |
| Zhu M | 2014 | Riboflavin-responsive multiple Acyl-CoA dehydrogenation deficiency in 13 cases, and a literature review in mainland Chinese patients | 10.1038/jhg.2014.10 |
| Béhin A | 2016 | Multiple acyl-CoA dehydrogenase deficiency (MADD) as a cause of late-onset treatable metabolic disease | 10.1016/j.neurol.2015.11.008 |
| Liu XY | 2016 | Skeletal muscle magnetic resonance imaging of the lower limbs in late-onset lipid storage myopathy with electron transfer flavoprotein dehydrogenase gene mutations | 10.4103/0366-6999.183423 |
| Angelini C | 2018 | Heterogeneous Phenotypes in Lipid Storage Myopathy Due to ETFDH Gene Mutations | 10.1007/8904_2017_27 |
| Zhao YW | 2018 | Muscle Magnetic Resonance Imaging for the Differentiation of Multiple Acyl-CoA Dehydrogenase Deficiency and Immune-mediated Necrotizing Myopathy | 10.4103/0366-6999.222323 |
| Hong DJ | 2019 | Clinical and muscle magnetic resonance image findings in patients with late-onset multiple acyl-CoA dehydrogenase deficiency | 10.1097/CM9.0000000000000032 |
| Nilipour Y | 2020 | Multiple acyl-coenzyme A dehydrogenase deficiency shows a possible founder effect and is the most frequent cause of lipid storage myopathy in Iran | 10.1016/j.jns.2020.116707 |
| Sun YM | 2020 | The clinical and genetical heterogeneity of riboflavin-responsive multiple acyl-coenzyme A dehydrogenase deficiency | 10.4274/balkanmedj.galenos.2022.2022-1-127 |
| Yildiz Y | 2020 | Determinants of Riboflavin Responsiveness in Multiple Acyl-CoA Dehydrogenase Deficiency | 10.1016/j.pediatrneurol.2019.06.015 |
| Yuan J | 2020 | Clinical, pathological, and genetic features of riboflavin responsive multiple acyl-CoA dehydrogenation deficiency caused by electron transfer flavoprotein dehydrogenase mutation | 10.1038/s10038-023-01216-3 |
| Ali A | 2021 | Clinical, Biochemical, and Genetic Heterogeneity in Glutaric Aciduria Type II Patients | 10.3390/genes12091334 |
| Kuo YC | 2021 | A systematic review of late-onset and very-late-onset multiple acyl-coenzyme A dehydrogenase deficiency: Cohort analysis and patient report from Taiwan | 10.1016/j.nmd.2021.01.006. |
| Staretz-Chacham O | 2021 | Multiple Acyl-CoA Dehydrogenase Deficiency with Variable Presentation Due to a Homozygous Mutation in a Bedouin Tribe | 10.3390/genes12081140 |
| Tang Z | 2021 | Clinical Presentations and Genetic Characteristics of Late-Onset MADD Due to ETFDH Mutations in Five Patients: A Case Series | 10.3389/fneur.2021.747360 |
| Liu HY | 2022 | Clinical characteristics and electron transfer flavoprotein dehydrogenase genetic mutations in 26 patients with riboflavin reactive lipid deposition myopathy | 10.1177/0300060520966499 |
| Lupica A | 2022 | Diagnostic Challenges in Late Onset Multiple Acyl-CoA Dehydrogenase Deficiency: Clinical, Morphological, and Genetic Aspects | 10.3389/fneur.2022.815523 |
| Wen B | 2022 | Clinical, pathological and genetic features and follow-up of 110 patients with late-onset MADD: a single-center retrospective study | 10.1093/hmg/ddab308 |
| Yamada K | 2022 | Clinical and molecular investigation of 37 Japanese patients with multiple acyl-CoA dehydrogenase deficiency: p.Y507D in ETFDH, a common Japanese variant, causes a mortal phenotype | 10.1016/j.ymgmr.2022.100940 |
| Zhang J | 2022 | Characterization of 31 Patients with Riboflavin-Responsive Multiple acyl-CoA Dehydrogenase Deficiency | 10.4274/balkanmedj.galenos.2022.2022-1-127 |
| Zheng W | 2022 | Features and diagnostic value of body composition in patients with late-onset multiple acyl-CoA dehydrogenase deficiency | 10.1007/s13760-022-01974-5 |
| Zhang HQ | 2023 | Hyperhomocysteinemia in patients with riboflavin-responsivemultiple acyl-CoA dehydrogenase deficiency | 10.1002/mus.27960 |
| Bilgin H | 2024 | The evaluation of inherited metabolic diseases presenting withrhabdomyolysis from Turkey: Single center experience | 10.1016/j.ymgmr.2024.101070 |
| Bisschof M | 2024 | Clinical, biochemical, and genetic spectrum of MADD in a South African cohort: an ICGNMD study | 10.1186/s13023-023-03014-8 |
| Schee JP | 2024 | Multiple Acyl-CoA Dehydrogenase Deficiency: Phenotypic and Genetic Features of a Malaysian Cohort | 10.3988/jcn.2023.0265 |
