# Supplementary material for: The difference of variation types between late-onset multiple acyl-CoA dehydrogenase deficiency patients carrying biallelic and single heterozygous variations in ETFDH: a systematic review and meta-analysis
Source: Orphanet J Rare Dis. 2025 Jun 18;20:310. doi: 10.1186/s13023-025-03845-7 (PMC12178022; doi:10.1186/s13023-025-03845-7)
Supplement: Supplementary file 18 [file 13023_2025_3845_MOESM18_ESM.docx]

**Supplementary Table 5 Summary of pathogenicity prediction tools.**

| **Prediction tool** | **Score range** | **Deleterious threshold** | **Information used** |
| --- | --- | --- | --- |
| SIFT | 0 - 1 | ≤ 0.05 | Protein sequence homology and the physical properties of amino acids |
| PolyPhen-2 | 0 - 1 | ≥ 0.447 | Eight protein sequence features, three protein structure features |
| REVEL | 0 - 1 | ≥ 0.8 | An ensemble score based on 13 individual scores for predicting the pathogenicity of missense variants |
| MetaSVM | -2 - 3 | > 0 | Ensemble prediction scores incorporate 10 individual scores, alongside the maximum allele frequency observed in the 1000 Genomes populations |
| MetaLR | 0 - 1 | > 0.5 |  |
| MetaRNN | 0 - 1 | > 0.5 | An ensemble prediction score integrates 16 scores from various prediction tools, 8 conservation scores, and allele frequency data from the 1000 Genomes Project, ExAC, and gnomAD |

Legend: SIFT, Sorting Intolerant From Tolerant; PolyPhen-2, Polymorphism Phenotyping v2; REVEL, Rare Exome Variant Ensemble Learner; MetaSVM, Meta-Analytic Support Vector Machine; MetaLR, Meta-Analytic Logistic Regression; MetaRNN, Meta-Analytic Recurrent Neural Network.
