# Supplementary material for: The difference of variation types between late-onset multiple acyl-CoA dehydrogenase deficiency patients carrying biallelic and single heterozygous variations in ETFDH: a systematic review and meta-analysis
Source: Orphanet J Rare Dis. 2025 Jun 18;20:310. doi: 10.1186/s13023-025-03845-7 (PMC12178022; doi:10.1186/s13023-025-03845-7)
Supplement: Supplementary file 19 [file 13023_2025_3845_MOESM19_ESM.docx]

**Supplementary Table 4** JBI Critical Appraisal Checklist for included studies.

| Articles | Q1 | Q2 | Q3 | Q4 | Q5 | Q6 | Q7 | Q8 | Q9 | Q10 | %Yes | Risk |
| --- | --- | --- | --- | --- | --- | --- | --- | --- | --- | --- | --- | --- |
| Olsen RK 2007 | Y | Y | Y | Y | Y | Y | Y | Y | Y | NA | 90% | Low |
| Er TK 2010 | Y | Y | Y | U | Y | Y | Y | Y | Y | NA | 80% | Low |
| Lan MY 2010 | Y | Y | Y | Y | N | Y | Y | U | Y | NA | 70% | Low |
| Wang Y 2011 | Y | Y | Y | U | N | Y | Y | Y | Y | NA | 70% | Low |
| Wang ZQ 2011 | Y | Y | Y | Y | N | Y | Y | Y | Y | NA | 80% | Low |
| Xi JY 2011 | Y | Y | Y | Y | N | Y | Y | Y | Y | NA | 80% | Low |
| Zhu M 2014 | Y | Y | Y | Y | N | Y | Y | Y | Y | Y | 90% | Low |
| Béhin A 2016 | Y | Y | Y | U | Y | Y | Y | Y | Y | NA | 80% | Low |
| Liu XY 2016 | Y | Y | Y | Y | Y | Y | Y | N | Y | Y | 90% | Low |
| Angelini C 2018 | Y | Y | Y | U | Y | Y | Y | Y | Y | NA | 80% | Low |
| Zhao YW 2018 | Y | Y | Y | Y | N | Y | Y | N | Y | Y | 80% | Low |
| Hong DJ 2019 | Y | Y | Y | Y | Y | Y | Y | Y | Y | Y | 100% | Low |
| Nilipour Y 2020 | Y | Y | Y | Y | N | Y | Y | Y | Y | Y | 90% | Low |
| Sun YM 2020 | Y | Y | Y | Y | Y | Y | Y | Y | Y | NA | 90% | Low |
| Yildiz Y 2020 | Y | Y | Y | U | N | Y | Y | Y | Y | NA | 70% | Low |
| Yuan J 2020 | Y | Y | Y | Y | N | Y | Y | Y | Y | Y | 90% | Low |
| Ali A 2021 | Y | Y | Y | Y | N | Y | Y | N | Y | NA | 70% | Low |
| Kuo YC 2021 | Y | Y | Y | U | N | Y | Y | Y | Y | Y | 80% | Low |
| Staretz-Chacham O 2021 | Y | Y | Y | N | Y | Y | Y | Y | Y | NA | 80% | Low |
| Tang Z 2021 | Y | Y | Y | Y | Y | Y | Y | Y | Y | NA | 90% | Low |
| Liu HY 2022 | Y | Y | Y | Y | Y | Y | Y | Y | Y | NA | 90% | Low |
| Lupica A 2022 | Y | Y | Y | Y | Y | Y | Y | Y | Y | NA | 90% | Low |
| Wen B 2022 | Y | Y | Y | Y | N | Y | Y | Y | Y | NA | 80% | Low |
| Yamada K 2022 | Y | Y | Y | Y | N | Y | Y | Y | Y | NA | 80% | Low |
| Zhang J 2022 | Y | Y | Y | Y | N | Y | Y | Y | Y | NA | 80% | Low |
| Zheng W 2022 | Y | Y | Y | Y | N | Y | Y | Y | Y | Y | 90% | Low |
| Zhang HQ 2023 | Y | Y | Y | Y | N | Y | Y | Y | Y | Y | 90% | Low |
| Bilgin H 2024 | Y | Y | Y | Y | N | Y | Y | N | Y | Y | 80% | Low |
| Bisschof M 2024 | Y | Y | Y | Y | N | Y | Y | Y | Y | NA | 80% | Low |
| Schee JP 2024 | Y | Y | Y | Y | Y | Y | Y | Y | Y | Y | 100% | Low |

Legend: JBI, Joanna Briggs Institute; Q, question; “Y” indicates yes, “N” indicates no, “U” indicates unclear, “NA” indicates not applicable.

| Q1. Were there clear criteria for inclusion in the case series? |
| --- |
| Q2. Was the condition measured in a standard, reliable way for all participants included in the case series? |
| Q3. Were valid methods used for identification of the condition for all participants included in the case series? |
| Q4. Did the case series have consecutive inclusion of participants? |
| Q5. Did the case series have complete inclusion of participants? |
| Q6. Was there clear reporting of the demographics of the participants in the study? |
| Q7. Was there clear reporting of clinical information of the participants? |
| Q8. Were the outcomes or follow-up results of cases clearly reported? |
| Q9. Was there clear reporting of the presenting site(s)/clinic(s) demographic information? |
| Q10. Was statistical analysis appropriate? |
